# Evaluation of Data-Driven Rigid Motion Correction in Clinical Brain PET Imaging

**DOI:** 10.2967/jnumed.121.263309

**Published:** 2022-10

**Authors:** Matthew G. Spangler-Bickell, Samuel A. Hurley, Ali Pirasteh, Scott B. Perlman, Timothy Deller, Alan B. McMillan

**Affiliations:** 1 PET/MR Engineering, GE Healthcare, Waukesha, Wisconsin;; 2 Radiology, University of Wisconsin–Madison, Madison, Wisconsin; and; 3 Medical Physics, University of Wisconsin–Madison, Madison, Wisconsin

**Keywords:** PET, image reconstruction, data-driven motion correction, brain imaging

## Abstract

Head motion during brain PET imaging can significantly degrade the quality of the reconstructed image, leading to reduced diagnostic value and inaccurate quantitation. A fully data-driven motion correction approach was recently demonstrated to produce highly accurate motion estimates (<1 mm) with high temporal resolution (≥1 Hz), which can then be used for a motion-corrected reconstruction. This can be applied retrospectively with no impact on the clinical image acquisition protocol. We present a reader-based evaluation and an atlas-based quantitative analysis of this motion correction approach within a clinical cohort. **Methods:** Clinical patient data were collected over 2019–2020 and processed retrospectively. Motion was estimated using image-based registration on reconstructions of ultrashort frames (0.6–1.8 s), after which list-mode reconstructions that were fully motion-corrected were performed. Two readers graded the motion-corrected and uncorrected reconstructions. An atlas-based quantitative analysis was performed. Paired Wilcoxon tests were used to test for significant differences in reader scores and SUVs between reconstructions. The Levene test was used to determine whether motion correction had a greater impact on quantitation in the presence of motion than when motion was low. **Results:** Fifty standard clinical ^18^F-FDG brain PET datasets (age range, 13–83 y; mean ± SD, 59 ± 20 y; 27 women) from 3 scanners were collected. The reader study showed a significantly different, diagnostically relevant improvement by motion correction when motion was present (*P* = 0.02) and no impact in low-motion cases. Eight percent of all datasets improved from diagnostically unacceptable to acceptable. The atlas-based analysis demonstrated a significant difference between the motion-corrected and uncorrected reconstructions in cases of high motion for 7 of 8 regions of interest (*P* < 0.05). **Conclusion:** The proposed approach to data-driven motion estimation and correction demonstrated a clinically significant impact on brain PET image reconstruction.

## Visual Abstract

**Figure:**
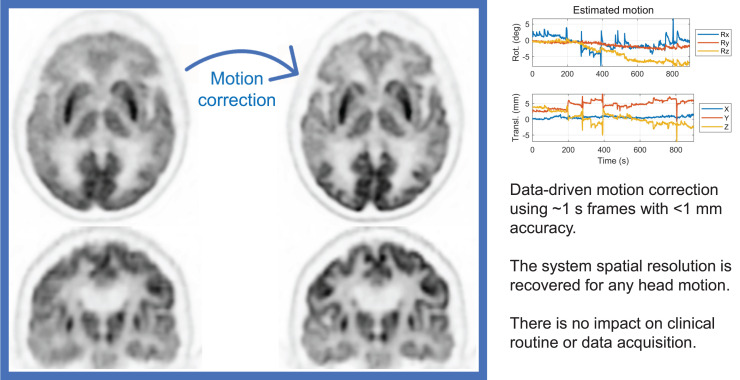

As the spatial resolution of modern whole-body PET scanners reaches 2–4 mm in full width at half maximum, together with improved sensitivity and time-of-flight resolution, it is becoming increasingly likely that even slight head motion may substantially degrade the reconstructed image. Although patient motion with translations of up to 15 mm and rotations of up to 4 degrees have been reported (1*,*^2^), less motion is quite common. Various motion-tracking and correction techniques have been presented for head motion (2–7). These usually use an external tracking device (such as a camera) to track a marker attached to the head (5), or they directly track the head (6). The motion estimates can then be used to perform frame-based reconstructions (8) or a full event-by-event motion-corrected (MoCo) reconstruction (9*,*^10^). However, none of these motion correction approaches have been implemented into widespread standard clinical routine, for several reasons. Some patient motion can be partially managed through head restraints and by discarding motion-corrupted portions of the data. To date, most motion-tracking methods rely on external hardware around the scanner (such as cameras) or attached to the patient (such as head markers), which complicate routine clinical protocols. Until recently, there has not been a substantial effort from vendors to incorporate motion correction into their products; efforts have thus remained predominantly within the research setting.

Fully data-driven approaches to motion correction that do not require external hardware have been presented. These usually estimate when motion occurred so that the data can be suitably framed (11), or they may estimate the motion itself to be used in a MoCo reconstruction (12–15). Because of the typically low count rates in PET imaging and long reconstruction times, the temporal resolution used for such motion estimation is usually on the order of tens of seconds or longer. Such low temporal resolutions may lead to residual intraframe motion blurring and inaccurate motion estimates. Alternatively, when higher temporal resolutions are used (on the order of ∼1 s), as described previously (12*,*^13^), the motion is estimated using centroid-of-distribution or inertial tensor calculations.

In this work, we evaluated a recently proposed approach to data-driven motion estimation and correction (16*,*^17^). The motion was estimated using rigid image registration on reconstructed images of very short frames. The estimated motion was then used in a full event-by-event MoCo list-mode reconstruction of the data, including all PET corrections. The approach is completely data-driven and can be applied retrospectively. An evaluation on 50 standard clinical ^18^F-FDG brain PET datasets is presented, showing the results of a reader study and an atlas-based quantitation analysis.

## MATERIALS AND METHODS

### Data

Patient data were acquired at the Wisconsin Institutes for Medical Research at the University of Wisconsin over 2019 and 2020, from a 4-ring Discovery MI PET/CT device (*n* = 11; 20-cm axial field of view), a Discovery 710 PET/CT device (*n* = 18), and a SIGNA PET/MRI device (*n* = 21) (all from GE Healthcare). Fifty consecutive ^18^F-FDG brain PET datasets were collected retrospectively, and none were rejected. All datasets were routine clinical imaging studies, and the need to obtain consent was waived by the institutional review board. Preliminary results using these datasets were presented at the 2021 annual meeting of the Society of Nuclear Medicine and Molecular Imaging (18); the current work presents a more thorough analytic and statistical evaluation.

### Motion Estimation and Image Reconstruction

**FIGURE 1.**
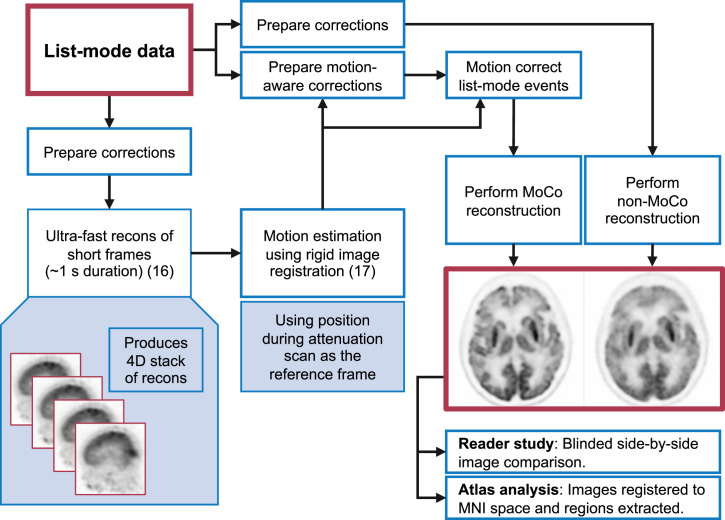
Flowchart of reconstruction process and analysis. 4D = 4-dimensional; MNI = Montreal Neurological Institute; recons = reconstructions.

The data were processed in 2 steps before being analyzed. Figure 1 shows a flowchart of the study. First, ultrafast reconstructions of very short frames (16) (0.6–1.8 s, set automatically and adjusted at each frame to ensure a constant 500 × 10^3^ true and scattered events per frame (17)) were performed for the entire scan duration, and image-based registration was then performed on these frames to estimate the motion. Rigid registration was performed using a least-squares metric and a gradient descent optimizer; further details were given in a previous publication (17). The 6 degrees of freedom of the motion were thus estimated directly at about 1 Hz, with an accuracy of less than 1 mm (measured as the mean error in absolute displacement of a mesh of points moved by the estimated motion) (17). The reference frame for the image registrations was chosen to ensure that the PET reconstruction aligned with the attenuation map. For PET/MRI, the MRI acquisitions for the attenuation map occur concurrently with the PET acquisitions; thus, the PET frames corresponding to data acquired during the MRI attenuation correction pulse sequence were averaged to create the reference frame. For PET/CT, however, the CT acquisitions occur before the PET acquisitions; thus, after estimating motion using the first PET frame as the reference, a single automatic cross-modality registration is performed using a mutual-information metric to set this reference frame to align with the CT image. The mean of all the short PET frames, after being aligned according to the estimated motion, was used for this registration. For 12 of the 29 PET/CT datasets, the automated cross-modality registration between the PET and the CT scans was suboptimal and manual intervention was required to ensure good registration. We will make this registration step more robust in the future to ensure fully automated processing.

The data were categorized into 4 motion groups using a metric based on the magnitude of the motion for each dataset. Similarly to previous investigators (19*,*^20^), we categorized the motion by moving points in image space and measuring their displacement. Although others have used an average displacement, we were interested in the maximum extent of the motion, and thus 2 points at the extreme extent of the brain were sufficient. Two points located in image space at 70 mm anterior and 70 mm posterior to the brain center were chosen and moved according to the estimated motion parameters. The brain center relative to the scanner center was set at a typically observed value for each scanner: for the PET/MRI device at 0, 40, and 20 mm for left–right, anterior–posterior, and superior–inferior, respectively (the anterior–posterior offset was due to the head coil used), and for both PET/CT devices at 0, 0, and 20 mm, respectively. The median absolute displacement from the reference was calculated for each point. The larger of these 2 medians was used as a metric to classify the datasets into 4 motion groups: low (median displacement < 1 mm), offset (median displacement < 1 mm but initial displacement > 2 mm), medium (median displacement = 1–2 mm), and high (median displacement > 2 mm).

The offset-motion group captures those datasets with little motion during the PET acquisition but with a large offset between the attenuation map acquisition and the PET acquisition (this usually applies only to PET/CT scans). This group classification was chosen empirically on the basis of our experience with many clinical datasets.

After estimation of head motion, a full reconstruction was performed, with each event being corrected according to the estimated motion, as shown in Figure 1. List-mode time-of-flight–based block sequential regularization expectation maximization (21*,*^22^) was performed with a β-parameter of 50. Spatially variant point-spread function modeling was performed using a hybrid image-space/projection-space approach (23). For clarity, the list-mode maximum likelihood expectation maximization with motion correction (9*,*^10^) is given here; subsets and a regularization term are added for the block sequential regularization expectation maximization:

$${\text{λ}}_{j}^{n+1}=\frac{{\text{λ}}_{j}^{n}}{{\bar{s}}_{j}}\overset{M}{\underset{m}{\sum }}c_{i_{m}^{\prime }\;j}\frac{1}{\sum_{k}c_{i_{m}^{\prime }k}{\text{λ}}_{k}^{n}+\frac{S^{\prime }{}_{i_{m}}+R_{i_{m}}}{a_{i_{m}^{\prime }}e_{i_{m}}{\text{σ}}_{i_{m}}}},$$

$${\bar{s}}_{j}=\overset{P}{\underset{p}{\sum }}w_{p}\overset{L}{\underset{l}{\sum }}c_{l_{p}^{\prime }j}a_{l_{p}^{\prime }}e_{l}{\text{σ}}_{l},$$

where ${\text{λ}}_{j}^{n}$ is the image value at pixel *j* and iteration *n*, $i_{m}$ is the line of response (LOR, the line joining a detecting crystal pair) *i* associated with list-mode event *m*, *M* is the total number of list-mode events, $i_{m}^{\prime }$ is the MoCo LOR *i* for event *m*, $c_{ij}$ is the system matrix, $S_{i}^{\prime }$ is the motion-aware scatter contribution along LOR *i*, $R_{i}$ is the randoms contribution, $a_{i}$ is the attenuation correction factor through the patient attenuation map along LOR *i*, $e_{i}$ is the attenuation correction factor through the attenuating material exterior to the patient along LOR *i*, and $\sigma_{i}$ is the scanner sensitivity factor (crystal efficiency and dead time) for LOR *i*. The time-averaged sensitivity image $\bar{s}$ is calculated by moving the endpoints of each LOR (*l*, of which there are *L* in the scanner) by a particular set of motion parameters *p*, backprojecting the appropriate attenuation and sensitivity factors, and calculating the time-weighted ($w_{p}$) average across all the motion data, *P*. The attenuation factors $a_{i}$ and $e_{i}$ are handled separately since the patient is moving whereas the rest of the attenuating material is not; therefore, the MoCo LORs ($i_{m}^{\prime }$) are used for the patient attenuation correction factors.

Additionally, a non-MoCo list-mode reconstruction was performed for comparison.

### Reader Study

The MoCo and non-MoCo reconstructions were randomized and read by a masked nuclear medicine physician with 36 y of experience and a dual–board-certified nuclear medicine physician and radiologist with fellowship training in nuclear medicine and body MRI with 2 y of experience. Images were evaluated on a 5-point Likert scale for sharpness and diagnostic quality, as specified in Table 1. The readers were not aware of the motion groups of the datasets; these were used only during the analysis.

**TABLE 1.** Likert-Scale Scoring for Image Evaluation

Score	Criteria	Diagnostic acceptability
1	Very poor	Nondiagnostic
2	Poor	Nondiagnostic
3	Acceptable	Diagnostic
4	Good	Diagnostic
5	Excellent	Diagnostic

### Quantitative Analysis

An atlas-based analysis of the quantitative accuracy of the reconstructions was performed. The reconstructions were individually and nonrigidly registered to an aggregated ^18^F-FDG atlas in Montreal Neurosciences Institute–152 image space (24*,*^25^) using the Advanced Normalization Tools toolbox (26). Eight regions of interest (ROIs) were extracted and analyzed: frontal lobe, occipital lobe, temporal lobe, parietal lobe, cerebellum, left/right cerebral cortex, and whole cerebral cortex.

### Statistical Analysis

For the reader study, interreader variability was tested using 2 metrics: the modified interrater agreement index ($r_{wg}^{*}$) was used to evaluate the 5-point Likert scores (27), and the Cohen κ (28) was used to evaluate agreement on whether an image was diagnostically acceptable (i.e., having a Likert score of ≥3). These tests were conducted for both reader questions and for the non-MoCo and MoCo reconstructions.

Using the average Likert scores for the 2 readers, paired Wilcoxon tests were conducted to test for significant differences between the median scores of the MoCo and non-MoCo reconstructions at a 2-sided significance level of *P* < 0.05, using the statistical toolbox in MATLAB (MathWorks). Correction was made for the false-discovery rate using the Benjamini–Hochberg procedure with a rate (Q-value) of 10%.

For the atlas-based quantitative analysis, paired Wilcoxon tests for significant differences between the medians of ROI SUV_max_ in the MoCo and non-MoCo reconstructions within each motion group were conducted at a 2-sided significance level of *P* < 0.05, with false-discovery-rate correction. Additionally, the relative differences between the SUV_max_ of the MoCo and non-MoCo reconstructions were calculated, and Levene tests (29) were conducted to determine whether the variance in these differences for each motion group was significantly different from the low-motion group, indicating that motion correction had made a quantitative difference in reconstructions when motion was present. A 2-sided significance level of *P* < 0.05 was used, and false-discovery-rate correction was applied.

## RESULTS

### Patient Characteristics and Injected Activity

Patient age ranged from 13–83 y (mean ± SD, 59 ± 20 y). There were 27 women. The injected activity was 390 ± 24 MBq for the Discovery 710 (55 ± 4 min uptake time, 15-min duration, 4.6 ± 1.1 × 10^8^ counts), 400 ± 26 MBq for the Discovery MI (56 ± 5 min uptake time, 15-min duration, 8.9 ± 2.3 × 10^8^ counts), and 450 ± 85 MBq for the SIGNA (63 ± 13 min uptake time, 25-min duration [except for 3 datasets with a 11- to 15-min duration], 17.2 ± 5.3 × 10^8^ counts).

### Motion Estimation

**FIGURE 2.**
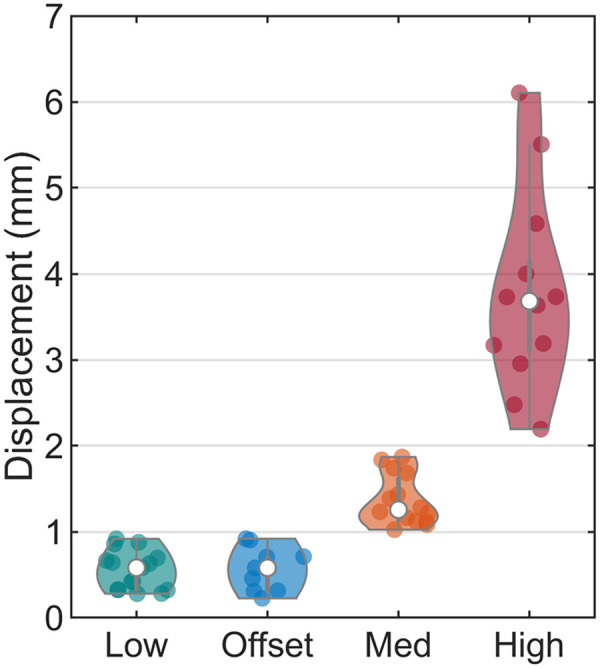
Violin plots (30) showing distribution of estimated motion for all datasets within 4 motion groups. Width of violins indicates density of data points, and length indicates range of data. Actual data points are scattered within violins, with white dot being median.

The number of datasets in each motion group is shown in Table 2, and the distribution of the displacement metric is shown in Figure 2.

**TABLE 2.** Distribution of Datasets Among Defined Motion Groups

Group	All scanners	Discovery 710	Discovery	Signa
Low	15 (30%)	4	1	10
Offset	9 (18%)	4	5	0
Medium	14 (28%)	6	4	4
High	12 (24%)	4	1	7

### Motion Correction Case Studies

**FIGURE 3.**
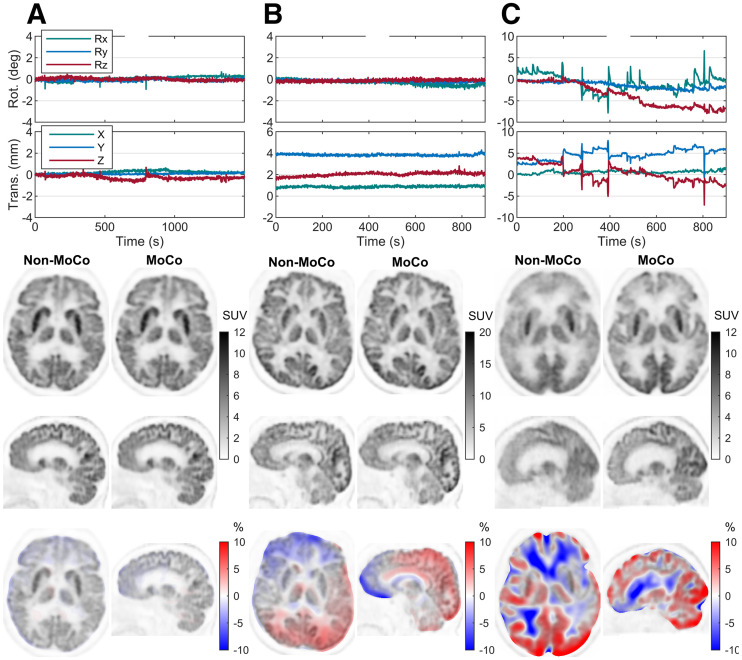
Example reconstructions for 3 case studies from low-motion (A), offset-motion (B), and high-motion (C) groups. All 3 are PET/CT data. Six degrees of freedom of motion data are plotted at top. Smoothed relative differences between images are shown at bottom overlayed on MoCo image. Low-motion case demonstrates that when there is little motion, motion correction has very small effect on reconstruction. Offset case shows that although no obvious differences are visible between images, relative-difference image shows gradient due to misalignment of Non-MoCo image with attenuation map. In high-motion case, much of blurring due to motion visible in Non-MoCo image has been removed in MoCo image. Rot. = rotation; Trans. = translation.

Figure 3 shows examples of reconstructions from the low-, offset-, and high-motion groups. The relative-difference images were calculated as (MoCo − non-MoCo)/MoCo × 100%.

### Reader Study

**FIGURE 4.**
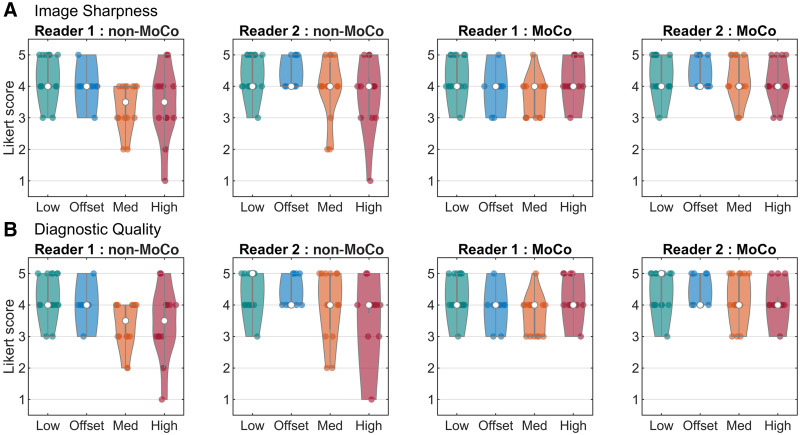
Likert scores for MoCo and non-MoCo reconstructions for 2 questions: image sharpness (A) and diagnostic quality (B). In non-MoCo cases, reader scores had more variation among datasets in higher-motion groups, with some images not being diagnostically acceptable. In MoCo cases, scores were consistent across all motion groups.

The results of the interreader variability analysis are shown in Table 3. Agreement between the readers was high according to all tests. Notably, agreement on whether an image was diagnostically acceptable (κ) was higher (no disagreement) for the MoCo reconstructions than for the non-MoCo reconstructions (for which there was 1 disagreement). The Likert scores for the 2 readers are shown in Figure 4 for the 2 questions. In 5 (10%) of the 50 datasets, the diagnostic quality of the reconstruction with motion correction improved by at least 1 on the Likert scale. The non-MoCo reconstructions for 4 (8%) datasets were rated as not diagnostically acceptable, and for all of these the MoCo reconstructions were rated as diagnostically acceptable. The results of the paired Wilcoxon tests on the reader scores are shown in Table 4.

**TABLE 3.** Results of Interreader Variability Analysis (High Agreement in All Cases)

	Non-MoCo		MoCo	
Parameter	$r_{wg}^{*}$	κ	$r_{wg}^{*}$	κ
Image sharpness	0.98	0.85	0.98	1
Diagnostic quality	0.98	0.85	0.98	1

**TABLE 4.** *P* Values of Paired Wilcoxon Tests Between MoCo and Non-MoCo Reconstructions, According to Reader Likert Scores

Motion group	Low	Offset	Medium	High	All*
Image sharpness	>0.99	>0.99	0.06	0.02^†^	0.003^†^
Diagnostic quality	>0.99	>0.99	0.13	0.02^†^	0.003^†^

* All motion groups considered together.

† Significant difference (*P* < 0.05, false-discovery-rate–corrected).

### Quantitative Analysis

**FIGURE 5.**
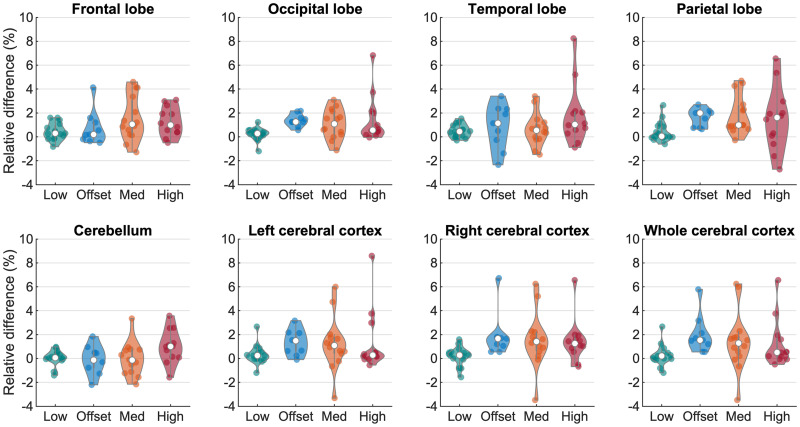
Relative differences between SUV_max_ of ROIs extracted from MoCo and non-MoCo reconstructions. Differences are larger in higher-motion groups, as expected. Since MoCo reconstruction ensures better alignment with attenuation map, SUV_max_ is expected to be more accurate in MoCo reconstructions than in non-MoCo reconstructions, regardless of which is greater or lesser.

The relative differences in ROI SUV_max_ between the MoCo and non-MoCo reconstructions are shown in Figure 5, calculated as (MoCo SUV_max_ − non-MoCo SUV_max_)/MoCo SUV_max_ × 100%. Table 5 presents the results of the statistical analysis.

**TABLE 5.** Results from Paired Wilcoxon Tests on ROI SUV_max_ of MoCo and Non-MoCo Images and from Levene Tests on Variance of ROI SUV_max_ Relative-Difference Values

	Wilcoxon test on SUV_max_				Levene test on relative difference in SUV_max_		
Site	Low	Offset	Medium	High	Offset	Medium	High
Frontal lobe	0.03*	0.25	0.009*	0.007*	0.10	0.002*	0.02*
Occipital lobe	0.03*	0.004*	0.009*	0.001*	0.73	0.001*	0.005*
Temporal lobe	0.007*	0.16	0.10	0.007*	0.001*	0.04*	0.01*
Parietal lobe	0.11	0.004*	<0.001*	0.08	0.89	0.01*	0.009*
Cerebellum	0.39	>0.99	0.71	0.03*	0.05	0.04*	0.04*
Left cerebral cortex	0.09	0.008*	0.02*	0.03*	0.20	0.0499*	0.01*
Right cerebral cortex	0.21	0.004*	0.01*	0.009*	0.24	0.07	0.24
Whole cerebral cortex	0.21	0.004*	0.03*	0.02*	0.13	0.04*	0.03*

* Significant difference (*P* < 0.05, false-discovery-rate–corrected).

Figure 5 shows that the relative differences in SUV_max_ between the MoCo and non-MoCo reconstructions were larger in the higher-motion groups than in the low-motion group. In the high-motion group, the SUV_max_ of the parietal lobe in the MoCo reconstructions differed from that in the non-MoCo reconstructions by 1.5% ± 2.7%, with a maximum of 6.6%, and in the temporal lobe the SUV_max_ differed by 1.8% ± 2.6%, with a maximum of 8.2%. In all cases, the SUV_max_ in the MoCo reconstruction is assumed to be more accurate, whether it was higher or lower than the non-MoCo reconstruction, since the former ensures better alignment with the attenuation map and has reduced motion blurring. Table 5 demonstrates that in the high-motion group the SUV_max_ significantly differed between the MoCo and non-MoCo reconstructions in 7 of 8 ROIs. Even in the offset-motion group, in which there was minimal motion during the PET acquisition, motion correction made a significant difference in 5 of 8 ROI SUV_max_ results because of the improved alignment with the attenuation map. The Levene tests indicated that the variance in relative differences between the MoCo and non-MoCo reconstructions was higher for 7 of the 8 ROIs in the medium- and high-motion groups than in the low-motion group (Table 5). These variances can be seen in the extent of the plots in Figure 5, comparing the higher-motion groups with the low-motion group. Table 5 indicates that when there was high motion, the motion correction significantly changed the reconstruction. The results of the reader study then confirm that the MoCo reconstructions were preferred.

## DISCUSSION

An evaluation of a fully data-driven motion estimation and correction technique for reconstruction of brain PET datasets has been presented. Fifty standard clinical ^18^F-FDG brain PET datasets were processed retrospectively, acquired on 1 PET/MRI and 2 PET/CT scanners. No additional motion-tracking hardware was used during the scan, and there was no impact on the standard clinical routine. The motion estimation used a temporal resolution of about 1 s and detected motion of more than 1 mm in 70% (35/50) of cases and more than 2 mm in 24% (12/50) of cases, the latter of which usually resulted in visually obvious differences between the MoCo and non-MoCo reconstructions. The masked reader study showed that the MoCo reconstructions improved the diagnostic quality in 10% (5/50) of the datasets, and the improvement was significant in the high-motion group (*P* = 0.02) and when considering all the data (*P* = 0.003). In 8% (4/50) of the datasets, which is a substantial portion of the cohort, the image was improved from diagnostically unacceptable to acceptable. The atlas-based quantitative analysis found significant differences (*P* < 0.05) in the SUV_max_ in 7 of the 8 ROIs in the medium- and high-motion groups and no significant differences in 5 of 8 ROIs in the low-motion group. The reader study confirmed that the image quality of the MoCo reconstructions was preferred over the non-MoCo reconstructions when motion was present and did not affect the scores when no motion was present, while the atlas-based analysis confirmed that motion correction does affect the quantitation of the reconstructions in the presence of motion.

Our study incorporated data from 3 scanners with very different geometries, with the axial field of view ranging from 157 to 250 mm, and all scanners benefitted from motion correction. The higher sensitivity and time-of-flight resolution of modern scanners allows for use of shorter frame durations for motion estimation and, hence, improved temporal sampling. To optimize temporal sampling, scanner-specific optimization may be necessary (17). Motion estimation and reconstruction were performed in a research setting and took approximately 2 h, which was about 30% longer than the non-MoCo reconstruction of the same dataset. Significantly faster reconstruction is expected with software optimization and dedicated hardware (e.g., graphics processing units) to ensure that the approach can be clinically feasible in future work.

This study had some limitations. We focused on ^18^F-FDG because it is the most common clinically used radiotracer. However, assuming that accurate motion estimates can be obtained with other radiotracers, we expect that motion correction would have a similar effect on reconstructions of such datasets. Accurate motion estimation has been demonstrated previously with ^18^F-florbetaben using this approach (17). Optical motion tracking was not available for comparison, as the data were processed retrospectively, and such a comparison was not the intention of this work. This work did not include examinations for which the activity distribution of the radiotracer inside the brain may change substantially during the scan, such as an ^15^O-H_2_O brain perfusion study, since the reference frame used for registration would not be representative of the entire dataset. Although an approach for motion estimation in such cases would be more challenging and was outside of the scope of the current work, we believe that a data-driven solution is possible; such a solution is the topic of ongoing research. Lastly, whereas image quality before and after motion correction was evaluated, the diagnostic implications of the MoCo images were not fully investigated. Considering the promising nature of our current results, we plan to further investigate the clinical impact of the application.

## CONCLUSION

We have presented an evaluation of a data-driven technique for correction of head motion in brain PET imaging. We demonstrated that motion is prevalent among standard clinical datasets and that motion correction has a significant impact on reconstructions, both qualitatively and quantitatively. The application of motion correction was not detrimental to image quality or quantification when no motion was present. Because motion is a known confounder of clinical brain PET, using data-driven motion correction will likely have important implications for diagnostic and research studies in which motion may occur. Given that the proposed solution relies entirely on retrospective reconstruction, it can readily be adopted into routine PET imaging procedures.

## Notes

KEY POINTS **QUESTION:** How effective is the proposed data-driven head motion correction technique? **PERTINENT FINDINGS:** Motion was observed in 70% of the study cohort, consisting of 50 consecutively acquired datasets. A reader study showed that all datasets that were deemed diagnostically unacceptable without motion correction (8%, 4/50) were then diagnostically acceptable with motion correction, with a significant improvement in cases of high motion. An 8-ROI atlas-based quantitative analysis concluded that motion correction had a significant impact on SUV_max_ (≤9%) in 65% of the ROIs. **IMPLICATIONS FOR PATIENT CARE:** Patient motion would no longer be a concern for ^18^F-FDG PET brain imaging when using this technology; patients could be scanned regardless of motion risk, and any motion would be corrected.
